# Pheochromocytoma of the urinary bladder

**DOI:** 10.1590/0100-3984.2015.0204

**Published:** 2017

**Authors:** André Martins Fernandes, Bernardo Vieira Paim, Ana Paula Aguiar Vidal, Edson Marchiori, Daniella Braz Parente

**Affiliations:** 1 Universidade Federal do Rio de Janeiro (UFRJ), Rio de Janeiro, RJ, Brazil.; 2 Instituto D'Or de Pesquisa e Ensino, Rio de Janeiro, RJ, Brazil.

**Figure 1:**
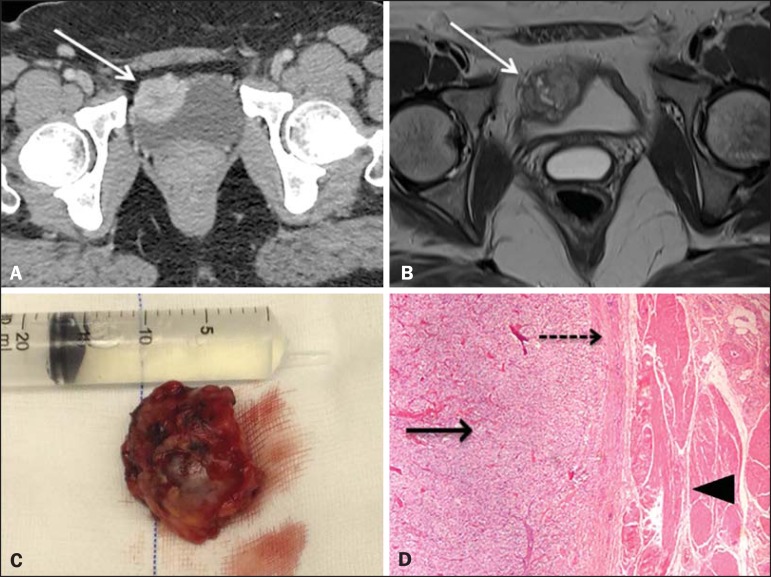
**A:** Intravenous contrastenhanced axial CT scan showing a hypervascular nodule in the anteroinferior wall of the bladder (arrow). **B:** T2-weighted MRI sequence showing a lesion with an isointense signal at the same site, with a heterogeneous signal and foci of hyper intense signals in its center (arrow). **C:** Surgical specimen (resection of the lesion). **D:** Hematoxylin and eosin-stained histological section, showing a lesion with a standard zellballen (nested) pattern (solid arrow), tumor capsule (dashed arrow), and the bladder wall (arrowhead).

Dear Editor,

A 44-year-old female presented with a 7-year history of paroxysmal episodes of dyspnea, headache, palpitation, tremors, and hypertension. In each episode, there had been a sudden onset of symptoms, with no triggering factors, and spontaneous improvement after approximately 15 min. On physical examination, she presented no relevant findings or comorbidities. At hospital admission, she reported having had episodes of palpitation, tachycardia, and profuse post-micturition sweating, remaining asymptomatic between episodes. She was submitted to computed tomography (CT) and magnetic resonance imaging (MRI), as shown in Figures 1A and 1B, respectively. The CT scan, with intravenous administration of contrast medium, revealed a nodular lesion, measuring 3.5 × 3.0 cm, with lobulated contours and increased density in its soft parts, showing intense, heterogeneous enhancement, in the anteroinferior wall of the bladder. On MRI, the lesion presented a lobular pattern, with a heterogeneous signal on T2-weighted sequences, a predominance of isointense signals, and foci of hyperintense signals in its center. Surgical resection of the lesion (partial cystectomy) was performed. Examination of the surgical specimen, retrieved from the right anterior wall of the bladder, showed a yellowish tumor measuring 3.0 × 3.0 cm, with a macroscopic appearance similar to that of adrenal tissue (Figure 1C). The pathological examination of the specimen revealed extra-adrenal paraganglioma and tumor-free margins (Figure 1D). In the postoperative period and during the remainder of the hospital stay, the patient did not present any of the adrenergic symptoms previously reported.

Pheochromocytomas are tumors of the sympathetic nervous system and can be functioning or nonfunctioning, sometimes secreting catecholamines, thus causing paroxysmal hypertension, palpitations, headache, and syncope^(1)^. They are most common between the fourth and sixth decades of life. Approximately 10% are bilateral, 10% are malignant, 10% occur in children, and 10% are extra-adrenal. More than 90% are located in the adrenal gland, and 98% are intra-abdominal. Pheochromocytomas can occur anywhere from the base of the skull to the bladder; when located outside the adrenal gland, they are known as paragangliomas^(2)^. Pheochromocytoma of the urinary bladder is a rare tumor, originating from chromaffin cells of the sympathetic nervous system and located within the bladder wall, accounting for 0.06% of all bladder tumors and 6% of all paragangliomas^(3)^. In the bladder, it can produce symptoms typical of pheochromocytoma, including hematuria and micturition syncope resulting from the release of catecholamines by bladder contraction. In 10-15% of cases, paragangliomas of the bladder are nonfunctioning; another 10% show hormonal activity without clinical expression^(4)^. 

Recent studies have discussed the role of imaging examinations in the investigation of pelvic lesions^(5-10)^. The diagnostic imaging methods used in the investigation of pheochromocytomas include ultrasound, CT, MRI, and scintigraphy. For the detection of adrenal pheochromocytomas > 1.0 cm in diameter, CT and MRI have a sensitivity of nearly 95% and 100%, respectively, and MRI has greater specificity than does CT^(11)^. On MRI, pheochromocytoma typically manifests as an expansive lesion with low signal intensity on T1-weighted sequences and high signal intensity on T2-weighted sequences, with intense impregnation after contrast administration. However, in rare cases, pheochromocytoma can present low signal intensity on T2-weighted sequences^(2)^. The treatment of choice for paraganglioma is surgical resection, because most are benign and can be completely resected^(12)^.
